# Endoscopic ultrasonography compared with multidetector computed tomography for the preoperative staging of gastric cancer: a meta-analysis

**DOI:** 10.1186/s12957-017-1176-6

**Published:** 2017-06-02

**Authors:** Run-Cong Nie, Shu-Qiang Yuan, Xiao-Jiang Chen, Shi Chen, Li-Pu Xu, Yong-Ming Chen, Bao-Yan Zhu, Xiao-Wei Sun, Zhi-Wei Zhou, Ying-Bo Chen

**Affiliations:** 1 Sun Yat-sen University Cancer Center; State Key Laboratory of Oncology in South China; Collaborative Innovation Center for Cancer Medicine, 651 E Dongfeng Road, Guangzhou, Guangdong 510060 China; 2 Department of Gastric Surgery, The 6th Affiliated Hospital, Sun Yat-sen University, Guangzhou, China

**Keywords:** Gastric carcinoma, Multidetector computed tomography, Endoscopic ultrasonography, Staging, Meta-analysis

## Abstract

**Background:**

The current study sought to perform a meta-analysis to compare the preoperative staging of endoscopic ultrasonography (EUS) and multidetector computed tomography (MDCT) in gastric carcinoma.

**Methods:**

Articles published between January 1, 2000, and April 1, 2016, that compared EUS with MDCT were included, and data were presented as 2 × 2 tables. The sensitivities, specificities and summary receiver operating characteristic (ROC) curves for T and N staging were calculated using a bivariate mixed effects model. Data were weighted by generic variance and then pooled by random-effects modeling.

**Results:**

Eight studies comprising 1736 patients were included in this meta-analysis. For T1 staging, the sensitivity value for EUS (82%) was significantly higher than that for MDCT (41%) (relative risk (RR): 2.06, 95% confidence interval (CI) 1.07–3.94; *P =* 0.030). For lymph node involvement, the sensitivity value for EUS (91%) was also significantly higher than that for MDCT (77%) (RR 1.14, 95% CI 1.05–1.23; *P =* 0.001). However, the specificity values of both EUS and MDCT were quite low, at 49 and 63%, respectively. No significant differences in T2–4 staging between EUS and MDCT were noted.

**Conclusion:**

This meta-analysis indicates that EUS may be superior to MDCT in preoperative T1 and N staging. Additionally, the low specificity values of EUS and MDCT for N staging merits attention.

## Background

On a worldwide scale, gastric cancer is the fourth most common malignancy and the third leading cause of cancer-related death [1]. With early diagnosis, more accurate preoperative staging and standardized curative surgery, gastric cancer patients receive more appropriate and less invasive treatment approaches, thus promoting an increase in overall survival [2–4]. Among these factors, accurate preoperative assessment of tumor invasion depth and lymph node metastasis is a fundamental first step in an optimal therapeutic approach. According to the new National Comprehensive Cancer Network (NCCN) practice guidelines for gastric cancer for clinical Tis or T1a gastric cancer patients, endoscopic mucosal resection (EMR) or endoscopic submucosal dissection (ESD) can be considered adequate therapy when the lesion is smaller than 2 cm without associated ulcer formation [5–7]. Results from two large phase III randomized controlled trials demonstrated that locally advanced gastric cancer patients could benefit from neoadjuvant chemotherapy [8, 9]. As a result, for patients who are diagnosed with clinical T2 disease or higher or with positive lymph node involvement, NCCN practice guidelines suggest that perioperative chemotherapy is a preferred treatment strategy.

Regarding the imaging for preoperative staging of gastric cancer, endoscopic ultrasonography (EUS) and multidetector computed tomography (MDCT) are the most commonly used techniques [10], especially in China. However, previous studies have reported conflicting results in preoperative staging between these two modalities [10–16]. Moreover, NCCN practice guidelines for gastric cancer do not recommend specific modalities or workup pathways [7]. Therefore, the purpose of our study was to perform a meta-analysis to compare EUS with MDCT for the preoperative staging of gastric carcinoma.

## Methods

### Search strategy

The PubMed and Web of Science databases were searched systematically for all relevant articles published between January 1, 2000, and April 1, 2016, that compared EUS with MDCT. The following key words were used in these literature searches: “EUS” OR “endoscopic ultrasound” OR “endosonography” OR “endoscopic ultrasonography” OR “computed tomography” OR “contrast-enhanced computed tomography” OR “multidetector computed tomography” and “stomach cancer” OR “gastric cancer” OR “gastric adenocarcinoma” and “sensitivity” OR “specificity” OR “accuracy” OR “diagnostic”. The reference lists of the articles retrieved were manually reviewed to identify additional relevant references.

### Study inclusion and exclusion criteria

Studies were included if they met the following criteria: (1) histologically proven gastric adenocarcinoma in more than 30 patients without any preoperative chemotherapy and/or radiation therapy; (2) histopathologic findings after gastrectomy used as the reference standard; and (3) data sufficient to construct a 2 × 2 contingency table. Studies were excluded based on the following exclusion criteria: (1) animals or ex vivo studies; (2) review articles, meta-analyses, abstracts, case reports, letters, and conference proceedings; and (3) studies published in a language other than English. The most recent or complete article was used if multiple articles described the same population.

### Data extraction

Data were extracted and summarized independently by two authors (RC, Nie and S, Chen). Two adjudicating senior authors (SQ, Yuan and YB, Chen) resolved any disagreement. The following data from each included study were extracted: first author, study design, year of publication, and sample size (number of patients).

### Study quality

Considering the lack of consensus to assess the quality of non-randomized clinical trials, studies used in this meta-analysis were selected based on data completeness and inclusion criteria.

### Statistical analyses

A bivariate mixed effects model was performed to acquire summary estimates of sensitivity and specificity and to fit summary receiver operating characteristic (ROC) curves. We used the relative risk (RR) for the comparison of the sensitivity and specificity of the two modalities. Our studies reported results with 95% confidence intervals (CIs). We used the *χ* ^2^ test to assess heterogeneity with the level of significance set at 10%, and the *I* ^2^ statistic was used to quantify heterogeneity.

The fixed-effects model was used if there was no significant heterogeneity between studies; otherwise, the random-effects model was performed [17]. We also used funnel plots to screen for publication bias if more than ten studies were included. Egger’s linear regression was used to test the effect of publication bias [18].

Analyses were performed using STATA SE 12.0 for Windows (StataCorp LP, College Station, TX).

## Results

### Study selection

**Fig. 1:**
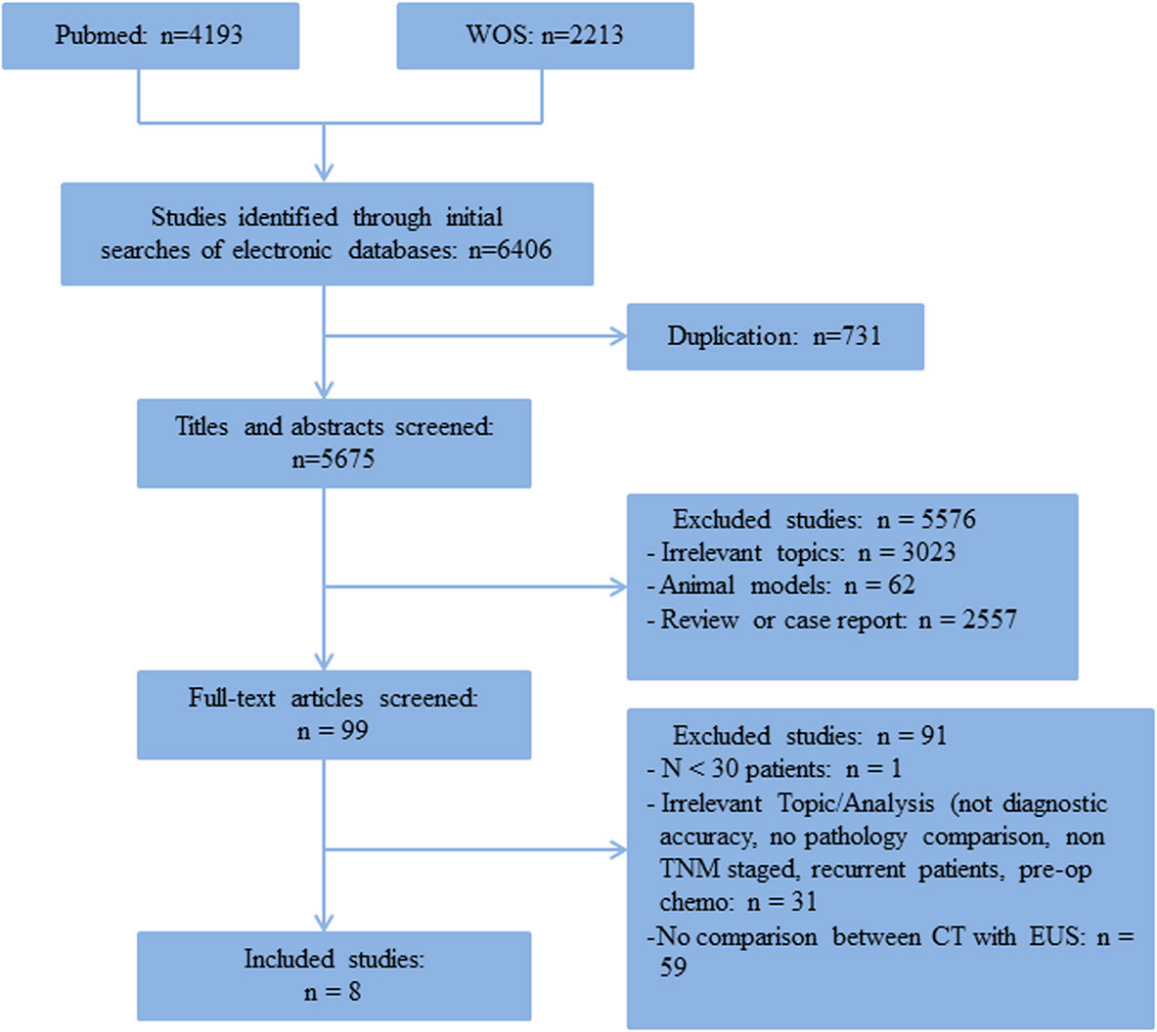
Flow diagram of the studies identified in the meta-analysis. MDCT, multidetector computed tomography; EUS, Endoscopic ultrasonography

A total of 5675 articles were identified using our search strategy. After screening their titles and abstracts, 5576 identical studies were excluded. The remaining 99 studies were fully reviewed. Ultimately, eight studies including 1736 cases were included for the final analysis [10–16, 19] (Fig. 1). The characteristics of the included studies are presented in Table 1. Among the eight studies, three were prospective studies [10–12], and five were retrospective studies [13–16, 19]. Agreement between the two reviewers was 97% for study selection.

**Table 1:** Characteristics of included studies

Study	Study type	No. of patient	Year	EUS MHz	Patient selection	Blinded	Reference test
Habermann et al.	R	51	2004	7.5/12.0	No	Yes	Yes
Polkowski et al.	P	88	2004	7.5/12.0	No	Yes	Yes
Ahn et al.	P	434	2009	5/12.0	Unknown	Yes	Yes
SW Hwang et al.	R	277	2010	5/7.5/12/20	No	Unknown	Yes
Furukawa et al.	R	175	2011	5/7.5/12/20	No	Yes	Yes
Feng et al.	R	610	2013	5/7.5/12/20	No	Yes	Yes
Fairweather et al.	R	49	2015	5/10	Unknown	Yes	Yes
Giganti et al.	P	52	2016	5/10	No	Yes	Yes

*R* retrospective study, *P* prospective study

### Summary estimates of sensitivity and specificity

The overall sensitivity and specificity of EUS and MDCT for gastric cancer patients are presented in Table 2.

**Table 2:** Sensitivity and specificity for EUS and MDCT imaging to diagnose T and N staging

Stage	Sensitivity (%)			Specificity (%)		
	EUS	MDCT	*P* value	EUS	MDCT	*P* value
T1	82 (64–92)	41 (13–77)	0.030	89 (52–98)	97 (80–100)	0.228
T2	72 (54–85)	48 (29–68)	0.056	84 (80–88)	86 (78–92)	0.629
T3	68 (52–80)	64 (37–84)	0.749	87 (77–93)	87 (70–95)	0.455
T4	52 (25–78)	61 (29–86)	0.613	97 (90–99)	97 (92–99)	0.731
*N*	91 (81–96)	77 (66–86)	0.001	49 (20–79)	63 (42–80)	0.079

*MDCT* multidetector computed tomography, *EUS* Endoscopic ultrasonography

#### T1 invasion

**Fig. 2:**
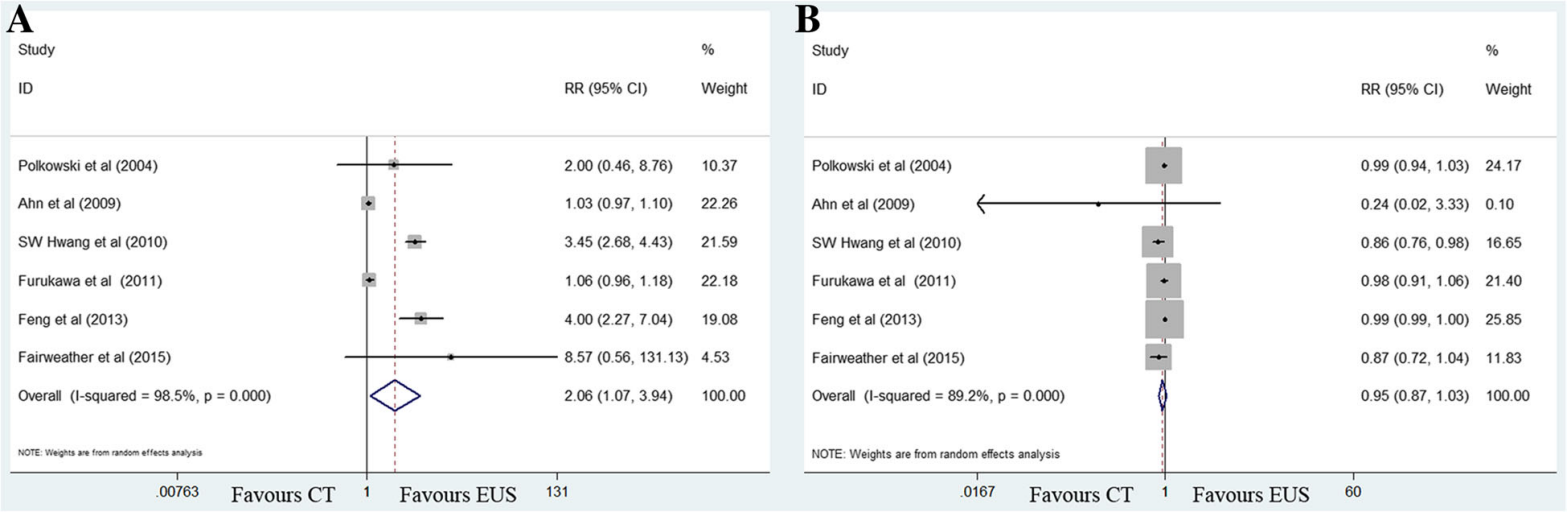
Forest plots of sensitivity (**a**) and specificity (**b**) for T1 staging. MDCT, multidetector computed tomography; EUS, Endoscopic ultrasonography. RR, relative risk; CI, confidence interval

The sensitivity value for EUS (82%) was significantly higher than that for MDCT (41%) (RR 2.06, 95% CI 1.07–3.94; *P =* 0.030), with significant between-study heterogeneity (*χ* ^2^ = 329.32, *P <* 0.001; *I* ^2^ = 98.5%). No significant publication bias was observed (*P =* 0.106). The specificity values for EUS and MDCT were 89 and 97%, respectively, and this difference was not significant (RR 0.95, 95% CI 0.87–1.03; *P =* 0.228) (Fig. 2).

#### T2 invasion

**Fig. 3:**
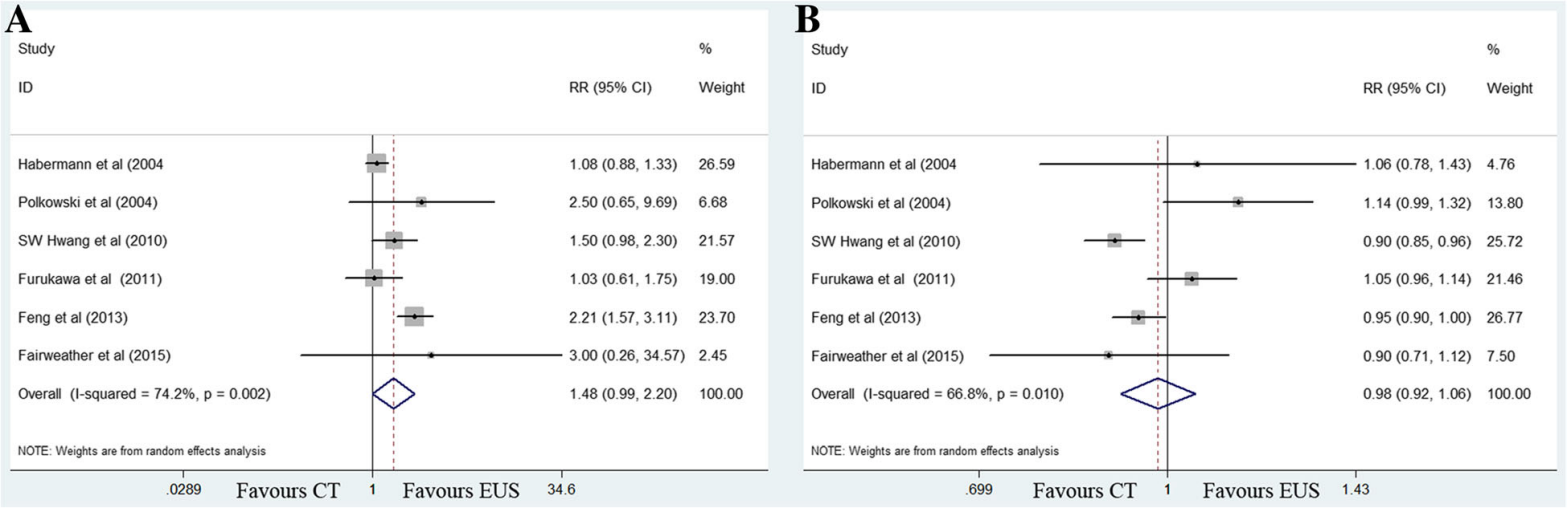
Forest plots of sensitivity (**a**) and specificity (**b**) for T2 staging. MDCT, multidetector computed tomography; EUS, Endoscopic ultrasonography. RR, relative risk; CI, confidence interval

The sensitivity value for EUS (72%) was marginally higher than that for MDCT (48%) (RR 1.48, 95% CI 0.99–2.20; *P =* 0.056), with significant between-study heterogeneity (*χ* ^2^ = 19.41, *P =* 0.002; *I* ^2^ = 74.2%). The specificity values for EUS (84%) and MDCT (86%) were similar (RR 0.98, 95% CI 0.92–1.06; *P =* 0.629) (Fig. 3).

#### T3 invasion

**Fig. 4:**
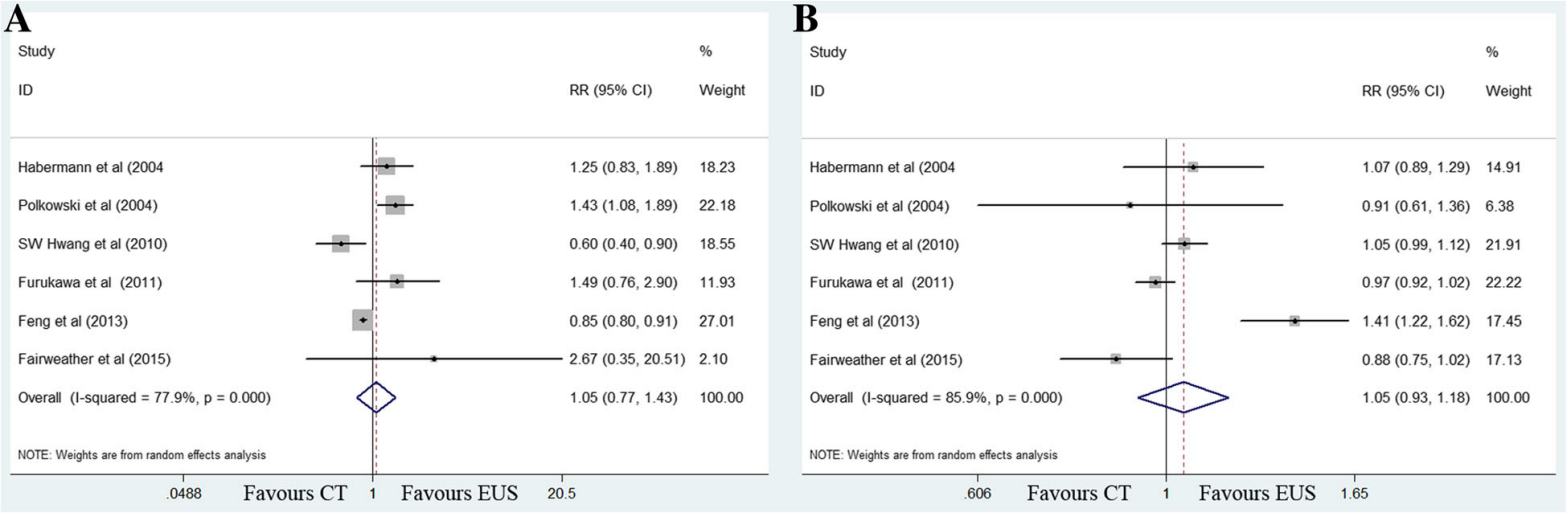
Forest plots of sensitivity (**a**) and specificity (**b**) for T3 staging. MDCT, multidetector computed tomography; EUS, Endoscopic ultrasonography. RR, relative risk; CI, confidence interval

No difference in sensitivity for EUS (68%) and MDCT (64%) was noted (RR 1.05, 95% CI 0.77–1.43; *P =* 0.749). EUS and MDCT imaging exhibited similar specificity estimates of 87% (Fig. 4).

#### T4 invasion

**Fig. 5:**
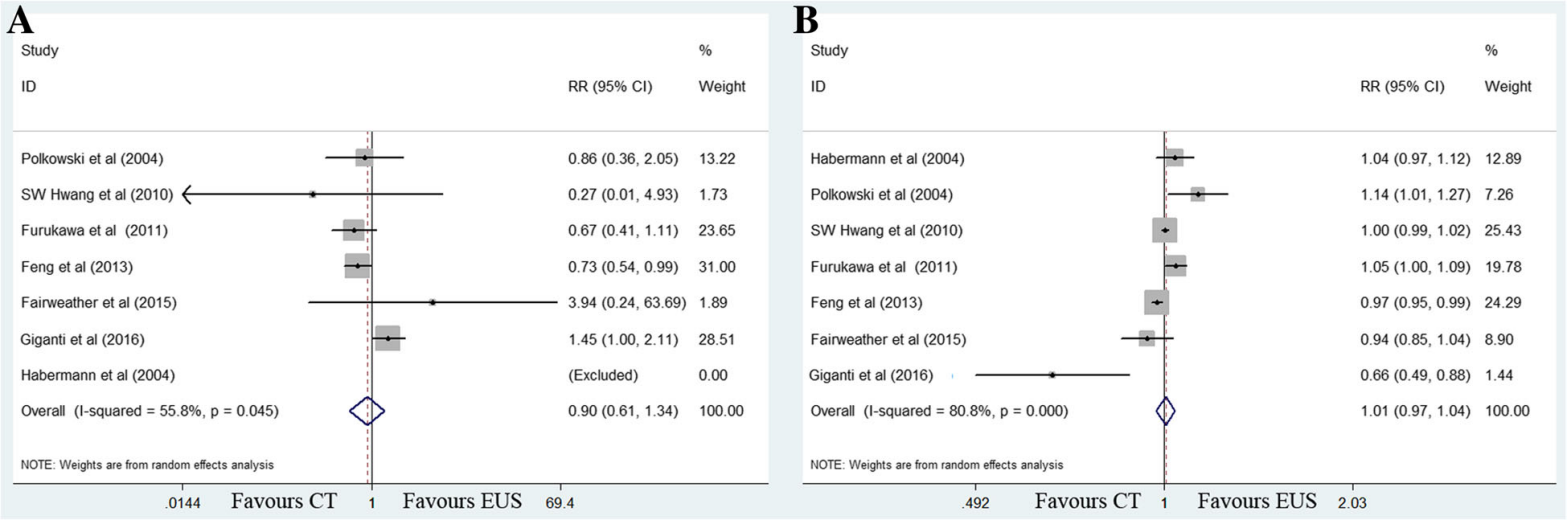
Forest plots of sensitivity (**a**) and specificity (**b**) for T4 staging. MDCT, multidetector computed tomography; EUS, Endoscopic ultrasonography. RR, relative risk; CI, confidence interval

Sensitivity estimates between the two imaging modalities were comparable: 52% for EUS and 61% for MDCT. Specificity values were also comparable: 97% for both EUS and MDCT (Fig. 5).

#### Lymph node involvement

**Fig. 6:**
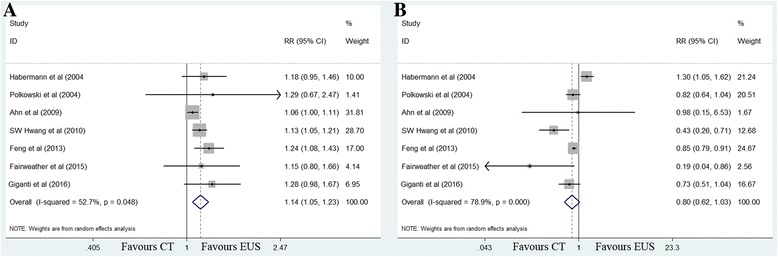
Forest plots of sensitivity (**a**) and specificity (**b**) for N staging. MDCT, multidetector computed tomography; EUS, Endoscopic ultrasonography. RR, relative risk; CI, confidence interval

The sensitivity value for EUS (91%) was significantly higher than that for MDCT (77%) (RR 1.14, 95% CI 1.05–1.23; *P =* 0.001), with moderate between-study heterogeneity (*χ* ^2^ = 12.68, *P =* 0.048; *I* ^2^ = 52.7%). No significant publication bias was observed (*P =* 0.073). Specificity was comparable between modalities, with values of 49% for EUS and 63% for MDCT (RR 0.80, 95% CI 0.62–1.03; *P =* 0.079) (Fig. 6).

### Summary ROC curves

**Fig. 7:**
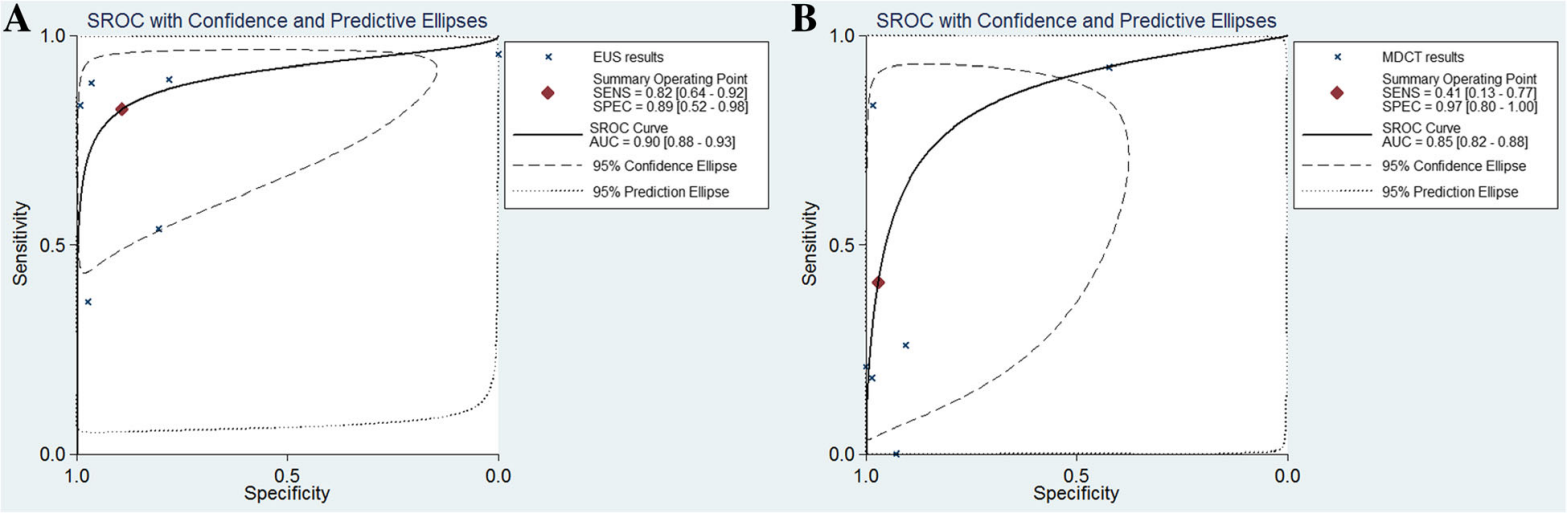
Summary ROC curves for T1 staging with EUS (**a**) and MDCT (**b**). MDCT, multidetector computed tomography; EUS, Endoscopic ultrasonography

**Fig. 8:**
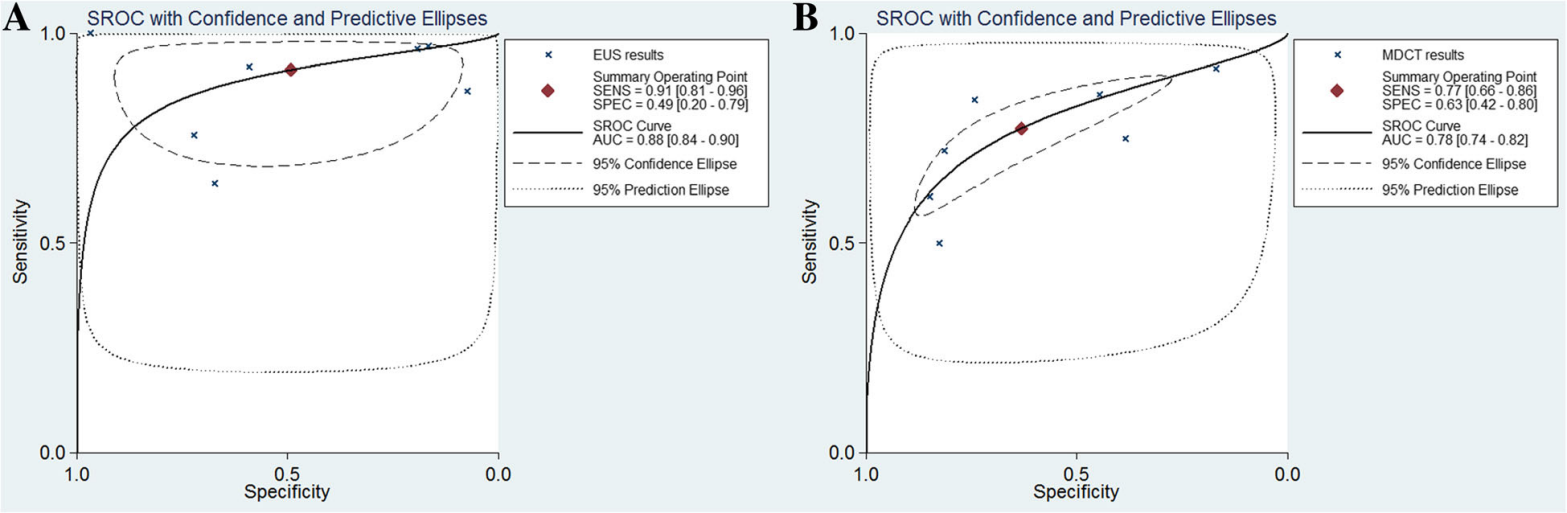
Summary ROC curves for lymph node involvement with EUS (**a**) and MDCT (**b**). MDCT, multidetector computed tomography; EUS, Endoscopic ultrasonography

Summary ROC curves of T1 invasion and lymph node involvement are presented in Figs. 7 and 8. A summary ROC curve located near the upper left corner with an increased area under the curve (AUC) indicates a better diagnostic modality. Summary ROC curves for T1 invasion using EUS were located closer to the upper left corner than those using MDCT, indicating the better diagnostic performance of EUS (Fig. 7). In addition, summary ROC curves demonstrated the better diagnostic performance of EUS than that of MDCT for lymph node involvement (Fig. 8).

## Discussion

Accurate preoperative evaluation for T and N staging of gastric cancer relies on precise imaging. Among various imaging modalities, EUS and computed tomography (CT) are the most common and valuable tools for the preoperative evaluation of gastric cancer. EUS was first introduced in clinical practice in the 1980s and exhibits high accuracy in detecting the depth of gastric cancer [20, 21]. Moreover, EUS shows greater accuracy than incremental CT for both the T and N staging assessment. However, MDCT exhibits a remarkably improved resolution and an accuracy and diagnostic performance similar to EUS [22–24]. Previous studies have reported conflicting results in preoperative staging between EUS and MDCT [10–16, 19]. This meta-analysis of three prospective studies and five retrospective studies including 1736 patients comparing the sensitivity and specificity of EUS and MDCT demonstrated that EUS was superior to MDCT in preoperative T1 and N staging. No significant differences in T2–4 staging were noted between EUS and MDCT.

With the increase in global aging, the proportion of gastric cancer patients older than 70 years is increasing, resulting in an increase in morbidity and mortality after curative gastrectomy. This trend has led to the need for less invasive treatment options, such as EMR and ESD, for early gastric cancer (EGC). Isomoto et al. demonstrated that 5-year overall and disease-specific survival rates after ESD reached 97.1 and 100%, respectively, indicating the excellent prognosis of ESD for EGC [6]. However, the standard imaging modality for clinical assessment of EGC remains debatable. Whether EUS is more accurate than MDCT in diagnosing EGC remains unknown [12, 15, 25]. This meta-analysis demonstrated that the sensitivity of EUS was significantly higher than that of MDCT for EGC, indicating that EUS was a preferred imaging modality in diagnosing EGC. For example, the EUS result was more reliable if a gastric cancer patient was preoperatively staged as T1 using EUS but T2 using MDCT. The reasons why EUS was superior to MDCT for T1 staging are as follows: (1) most EGC cases were easily treated by MDCT because EGC appeared to have small size without obvious enhancement of mucosa; and (2) the integrality of the low-density zone that corresponded to the submucosa was applied to distinguish T1 and T2. However, MDCT could easily distort the results in some cases due to edema or fatty deposition [26].

Feng et al. revealed that MDCT showed higher sensitivity than EUS with regard to lymph node metastasis [15], whereas other studies suggested comparable results in N staging [10, 11, 13, 19]. The pooled results of this meta-analysis demonstrated that EUS was superior to MDCT in preoperative N staging, with a higher sensitivity and better diagnostic performance. Our study revealed that EUS was more sensitive to recognize positive lymph node involvement and thus apply perioperative chemotherapy, which can reduce the tumor stage and significantly improved progression-free and overall survival [8, 9].

Notably, the pooled specificity values of EUS and MDCT were both quite low (49% for EUS and 63% for MDCT, respectively), indicating that the pooled value might not be reliable, even if EUS or MDCT suggested negative lymph node involvement. It is possible that in some cases, pathological positive lymph nodes may appear to be small without obvious enhancement. Zhao et al. also demonstrated that the rate of lymph node metastasis of EGC is quite high (25.27%), and EMR or ESD should be cautiously used in high-risk EGC patients [27]. Therefore, more accurate imaging modalities to predict the negative lymph node for EGC are needed, and further studies are required to test whether the combination of MDCT and EUS could improve the specificity of N staging.

There are also some limitations in the present meta-analysis. First, the main limitation is that most of the included studies were retrospective, with the exception of three prospective studies. Second, significant between-study heterogeneity was noted in this meta-analysis. Finally, the incorporation of various classification systems may have influenced the results of this meta-analysis. However, we applied strict criteria to include high-quality studies, and the use of random-effects modeling could adequately address the heterogeneity. In addition, T staging of all editions is similar; therefore, the comparison of pooled data was not influenced. Given that N staging varies in different editions, the present meta-analysis compared only the preoperative identification of N0 versus N+ disease of EUS and MDCT to make the included studies comparable.

## Conclusions

This meta-analysis indicates that EUS may be superior to MDCT in preoperative T1 and N staging. Additionally, the low specificity of both EUS and MDCT for N staging merits further attention.
